# Hæmorrhoids—Their Treatment by Cascara Sagrada

**Published:** 1884-06-20

**Authors:** F. C. Herr

**Affiliations:** Philadelphia, Pa.


### HÆMORRHOIDS—THEIR TREATMENT BY CASCARA SAGRADA.

By F. C. Herr, M.D., Philadelphia, Pa.

Every physician is aware of the intractable nature of many cases of hæmorrholds, and it is not infrequently that his best-directed efforts fail completely to afford that relief which is so much coveted by the sufferer. Except in those cases where surgical interference is demanded, it must not be assumed, despite the intensity of the symptoms, that a curative influence may not be effected by a judicious employment of drugs. While few cases which are non-surgical will remain unyielding to proper treatment, there are some, and no doubt they have been met by almost every physician of moderate practice. I allude to those cases which complicate cirrhotic disease of the liver, and where the portal and hæmorrhoidal obstruction becomes almost immovable, by reason of the mechanical interference with the blood current; much relief may be afforded in these cases, but therapeutics has not yet attained that perfection which enables us to cure them absolutely. There is yet another class of cases to which special attention may be called. They occur in the progress of the history of a case of chronic gastritis. The modus operandi of their production here is highly interesting. In chronic gastritis there is such a complete and wholesale perversion of the intestinal secretions (sometimes almost a suppression of them) that the bowel is deprived of its required stimulus, and consequently remains inactive. Its fecal contents are passed only after very great voluntary straining at stool, and this evacuation comes, perhaps, only once in several days. The primary link in the chain of causation here is the perverted intestinal secretion, and this demands early attention if we desire to relieve the patient from the distressing condition of hæmorrhoids. Let me say, en passant, that the materia medica affords no remedy that is comparable in efficiency to cascara sagrada for the restoration of abnormal intestinal secretion. In chronic gastritis it is invaluable, as it meets almost every indication without any deleterious influence that can be measured. Recently, a well-known physician in this city was hastily summoned to attend an acquaintance of the writer. His diagnosis was dyspepsia. We all know what that diagnosis means, or may mean, to the afflicted one—cardiac palpitation, dyspnœa, asthmatic paroxysms, etc., etc. In this particular case many of the worst phenomena were present. The attending physician, true to the traditions of the schools, interdicted all food, and ordered the following combinations of drugs to be taken, i. e.:

℞ Pulv. opii.....................................gr. iv,
Argent. nitrat...................................gr. iv.
M. Div. in pil. No. xx.

This is prescription number one. It really made the patient worse, A substitution followed. It was prescription No. 2, as follows:

℞ Pulv. opii .............................. gr. iv,
Mass. hydrarg.......................... gr. xxxij.
M. Div. in pil. No. viij.

Still the patient did not mend. This want of improvement was the signal for another change. It was made, and prescription No. 3, as follows, was given:

℞ Potass. bromid..................................
Potass. bicarb.................................
Tr. calumbæ.....................................
Infus. gent. comp..............................

I do not remember the quantity of each ingredient here, but the combination is correct. Still another prescription was given, which the patient could not take. All of these medicines were given in one week's time for dyspepsia. They did not cure the case, nor relieve the symptoms. The attending physician was discharged, and the writer was called in to see the patient. The diagnosis was confirmed, and with a proper regimen was conjoined the use of cascara cordial with most happy effects. That patient is not well, but under the changed treatment a very marked improvement has taken place. The intestinal functions have been greatly restored, while the entire digestive apparatus seems to have been much strengthened and invigorated. In a former publication the writer sought to direct especial attention to the value of cascara cordial in the management of dyspeptic disorders, and the case above narrated offers a fresh proof of this value. With this digression, we will return to the subject proper of this paper. The several classes of hæmorrhoids to which we have referred, namely, those which can be removed only by surgical procedure, those which complicate cirrhosis of the liver, and those which occur in the history of chronic gastritis, constitute by far the smaller number of cases which come under the observation of the physician. Perhaps few persons pass through life withont at some time or another becoming the subjects of hæmorrhoids, and it is to this larger class that we desire particularly to direct attention. It is to that variety (we allude to the etiological, not the pathological nature of the variety) of hæmorrhoids which readily yields to treatment that we wish to consider in relation to curative plans of therapeutic management. Whatever determines blood to the rectum tends to their production. It is manifest, therefore, that the employment of active cathartic medicines, all of which determine the blood to the rectum, may produce hæmorrhoids, and it is equally clear that such medicines are contraindicated in the hæmorrhoidal condition. A long line of drugs has been in vogue for their treatment, some useful, many more useless. The writer has had more decided benefit from the employment of the compound licorice powder of the Prussian Pharmacopœia than from any other single agent save one. The sine qua non of a cure is the solubility of the bowels. Without this not much can be confidently hoped for. With this, little is left to be done to facilitate a happy result. We said that any agent which directs the blood to the rectum is likely to aggravate, and even produce the hæmorrhoidal condition. An exception to this exists in the case of aloes. Despite the fact that it does produce congestion of the pelvic viscera, it is yet curative in piles. Barker's formulæ for its administration is as follows:

℞ Pulv. aloes soc., }

Saponis cast., } ........................... aa ℈ j,

Ext. hyoscyam ................................ ʒ ss,

Pulv. ipecac ................................... gr. v.

M. Div. in pil. No. XV.

The sulphides have been highly lauded in the treatment of piles. Stillingia has won deserved rank as an efficient agent in the cure of that variety of pile which is sequential to constipation. The "grape cure" has been successful in some cases. Iodoform, in suppository, has produced happy results. Ergot, in conjunction with nux vomica, will often effect a cure; but these remedies require, generally, the assistance of laxative agents, because, irrespective of free intestinal evacuation, an almost insuperable barrier stands in the way of permanent relief. During the past three months the writer has had a sufficient experience with the use of a new remedy in the management of hæmorrhoids to convince him that it possesses superior efficacy as a reliable curative agent in this distressing affection. It is not impossible that it may not afford relief in all varieties of the disorder; it is positively curative, however, in those forms which are dependent upon a constipated habit, and highly serviceable, as before indicated, in that variety which complicates chronic gastritis. Cascara sagrada, the remedy here referred to, has acknowledged merit in the treatment of chronic constipation. It is incomparably superior to any other agent known to the writer for the relief of this condition. It is, therefore, indicated in the treatment of hæmorrhoids as the solubility of the bowel becomes a matter of paramount importance. Nor is this all: usually, in these conditions, a complex state of affairs exists—the hæmorrhoids being, perhaps, one of the many expressions of this state. As constipation, habitual constipation we mean, is generally the result of perverted intestinal secretion and disordered digestive function, and as hæmorrhoids are generally the result of habitual constipation, it follows that a remedy which will correct the former conditions may cure the latter. Ample experience has shown that cascara sagrada is highly beneficial in intestinal sluggishness, and in the preparation known as cascara cordial its efficiency is heightened by a happy combination. This latter preparation, given, not in ten-minim doses, but in teaspoonful doses twice or thrice daily, will be found to exercise a speedy and happy action upon the hæmorrhoidal condition. The writer a few years ago prescribed rhamnus frangula with indifferent results in the same conditions mentioned here. It produced a sense of great uneasiness, and often griping. The rhamnus Purshiana is incomparably better, and in the preparation above noted it meets a demand which has for a long time existed in the medical profession, a demand for a remedy which would cure hæmorrhoids without subjecting the patient to any unpleasantness or inconvenience. To add to the list of remedies already in vogue one other for the treatment of piles would be something in a therapeutic sense, but to add one to the list which surpasses (as we are sure it will be found to do from extensive experience) all others in efficiency, is at least just cause for congratulation to the army of sufferers from hæmorrhoids.—*Ther. Gaz.*

